# 187. Nasopharyngeal Testing to Individualize Management of Acute Otitis Media

**DOI:** 10.1093/ofid/ofac492.265

**Published:** 2022-12-15

**Authors:** Thresia Sebastian, Mohammad Toseef, Melanie Kurtz, Holly M Frost

**Affiliations:** Denver Health and Hospital Authority, Denver, Colorado; Denver Health and Hospital Authority, Denver, Colorado; Denver Health and Hospital Authority, Denver, Colorado; Denver Health and Hospital Authority, Denver, Colorado

## Abstract

**Background:**

There are > 10 million antibiotics prescribed yearly for acute otitis media (AOM). The associated organism can influence the likelihood of antibiotic benefit and optimal treatment. A rapid diagnostic test (RDT) for AOM could prevent unnecessary antibiotic use, while assuring those likely to benefit from antibiotics receive the appropriate agent. Nasopharyngeal (NP) polymerase chain reaction can effectively exclude the presence of organisms in middle ear fluid and may be useful to individualize care.

**Objective:** To explore the potential cost-effectiveness and reduction in antibiotics with NP RDTs to direct AOM management.

**Methods:**

We developed two algorithms for AOM management based on NP bacterial otopathogens (*Figure1*). The algorithms provide recommendations on prescribing strategy (immediate, delayed, or observation) and antimicrobial agent. We utilized a tiering system based on expected pathogen-associated severity and resistance. The primary outcome was the incremental cost-effectiveness ratio (ICER) expressed as cost per quality adjusted life day gained (QALD). We used a decision-analytic model to evaluate the cost-effectiveness of the RDT algorithms compared to usual care from a societal perspective over a 30-day time horizon. Secondary outcomes included the (1) cost at which a RDT would be cost-effective and (2) potential reduction in annual antibiotics used.

Flow diagram

**Figure 1:**
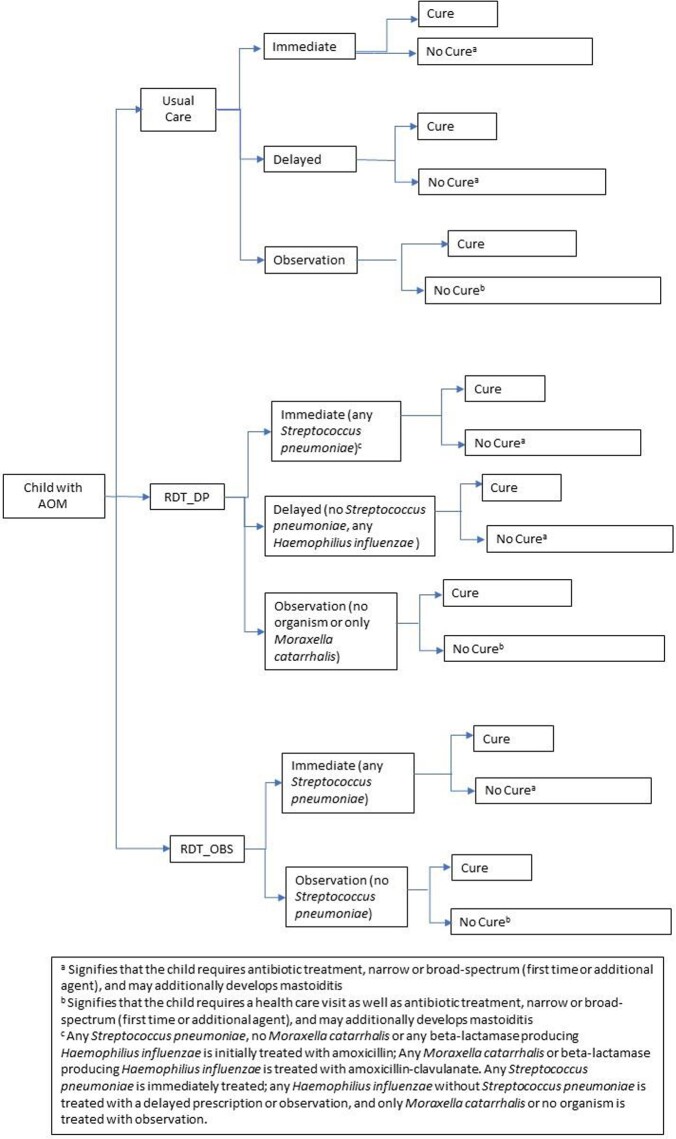
Flow diagram for management of AOM including RDT-DP and RDT-OBS algorithms

**Results:**

An RDT algorithm that used immediate, delayed prescribing, and observation based on pathogen (RDT-DP) had an ICER of $1336.15/QALD compared with usual care and strongly dominated the RDT that used only immediate prescribing and observation (RDT-OBS). At an RDT cost of $278.56, the ICER for RDT-DP exceeded the willingness to pay threshold ($274 per QALD gained); however, if the cost of the RDT was < $212.10, the ICER was below the threshold. Both algorithms reduced annual antibiotic use, including broad-spectrum use, compared to usual care (RDT-DP 4.7 million (56% reduction), RDT-OBS 5.4 million (49% reduction), usual care 10.5 million).

**Conclusion:**

The use of a NP RDT for AOM is likely to be cost-effective and substantially reduce unnecessary antibiotic use. These iterative algorithms could be modified to guide AOM management as scientific knowledge evolves.

**Disclosures:**

**All Authors**: No reported disclosures.

